# Challenges in Sheltering Seized Animals from Hoarders from a One Welfare Perspective

**DOI:** 10.3390/ani13213303

**Published:** 2023-10-24

**Authors:** Paola Fossati

**Affiliations:** Department of Environmental Science and Policy, Università degli Studi di Milano, Via Celoria 2, 20133 Milano, Italy; paola.fossati@unimi.it

**Keywords:** One Welfare, animal welfare, animal shelter, animal hoarding, hoarders, companion animals, seized animals, challenges

## Abstract

**Simple Summary:**

Animal hoarding is a serious behavioral issue that overwhelms hoarders and impacts many animals, causing them to suffer in inadequate, unsanitary, and hidden environments. It poses significant challenges to animal welfare, frequently leading to mistreatment, along with threats to human health. When such an obsessive accumulation of animals is discovered, exposing their harmful condition, there are not perfect solutions. Positive interventions can be planned by using the emerging One Welfare approach, which recognizes the interdependence of animal welfare, human well-being, and the environment. Typically, the accumulated animals are seized and relocated to a shelter, where additional challenges arise for both the animals and the shelter staff. The One Welfare approach, which is increasingly being used alongside One Health to work at the interface of human and animal health and welfare, could be adopted to address the poor state of humans while also planning strategies that benefit animals, including their conditions in shelters. In this paper, I examine the main issues surrounding animal hoarding, as well as the challenges raised by the common transfer of animals to shelters in light of the One Welfare principles.

**Abstract:**

Animal hoarding is a complex issue that, when discovered, frequently necessitates opening shelter doors to many animals. This is due to hoarders’ inability to provide even the most basic welfare standards for their animals, resulting in poor welfare conditions that frequently border on mistreatment. These people are frequently unaware of their failure to care for their animals, as well as of the harm that they cause to people around them and the environment. They usually do not care for themselves either. The majority of hoarders have difficult histories, and they all need help getting back on track. When the agencies discover the status quo, the animals are usually seized and taken to shelters, where they face a variety of welfare consequences, beginning with confinement in an unknown environment that is associated with additional risks (e.g., infectious diseases, behavioral deterioration, and distress). Furthermore, the targeted shelters are frequently overcrowded and cannot adequately accommodate the large numbers of animals found in hoarders’ environments. The One Welfare approach, which is increasingly being used alongside One Health to work at the intersection of human and animal health and welfare, could be adopted to benefit animals while also addressing the poor states of humans. This concept’s depiction of the interconnections between animal welfare, human well-being, and the environment can fit with all the components of the animal-hoarding phenomenon, including the peculiarities of the hoarding environment, as well as those of shelters where animals are often moved. The purpose of this paper is to offer insights into how the One Welfare concept may be critical in tackling all of the interests concerned in these cases and offering solutions.

## 1. Introduction

Animal welfare is a concept that is becoming increasingly rich in meaning not only as knowledge about animals’ sentience progresses, but also as evidence of the direct and indirect links between animal welfare and human welfare, as well as between the latter and the environment [1,2].

This link is evident in various contexts of social life, particularly those in which welfare levels are directly proportional to one another: better human welfare corresponds to better animal welfare, and vice versa [3].

The plight of animal hoarders is one example of the deteriorating living conditions of both animals and the people who keep them in their homes. Animal hoarding is a behavioral problem that leads some people to the compulsive need to keep a higher-than-usual number of animals, often to the point that they become overwhelmed by such an accumulation and are unable to provide even minimal standards of care for either the animals or themselves. Many situations develop from predisposing psychosocial factors, like psychopathology, stress, and loneliness [4,5,6]. The consistency of the phenomenon of animal hoarding is actually unknown, as it has been and continues to be poorly researched, studied, and described in the scientific literature, despite being found in many communities around the world [7,8,9,10]. It could be argued that animal hoarding follows similar trends of distribution across nations and cultures. Nevertheless, there is still a need to raise awareness about this condition and obtain more data on animal hoarding. Authors dealing with the subject have remarked that conducting the epidemiology is difficult because the casuistry is not always coherently articulated and access to case reports is frequently limited due to privacy issues [11]. Furthermore, “hoarding disorders” were only listed as a new diagnosis in the Fifth Edition of the American Diagnostic and Statistical Manual of Mental Disorders (DSM-5), published in 2013 [12], whereas the International Classification of Diseases (ICD), which is the standard diagnostic tool for epidemiology, health management, and clinical purposes, mainly used in the UK and Europe, listed it in the 11th Revision of the International Classification of Diseases (ICD-11), which officially came into effect on 1 January 2022 [13]. Of note, despite the fact that the DSM-5 contains a brief description of animal-hoarding disorder (basically referring to the accumulation of a large number of animals and a failure to provide minimal standards of any kind of care and to avoid the deteriorating condition of the animals and the environment), in both the DSM-5 and ICD-11 diagnostic criteria to date, the object of accumulation has not been identified as a discriminating diagnostic criterion. Therefore, people who accumulate animals are not distinguished from those who accumulate inanimate items. It should also be considered that several cases of animal-hoarding behavior may go unnoticed because they are hidden or are seen as an altruistic gesture that is socially acceptable rather than abnormal.

In this context, nationally representative data on the prevalence of hoarding disorders are neither available nor updated. Recent systematic reviews of animal-hoarding disorders indicate that the related behavior is observed in an average of 2–6% of the population in the United States and Europe [10,11,14,15]. This point prevalence of clinically severe hoarding in the United States and Europe is also reported in the DSM-5.

Unfortunately, the lack of data also applies to shelters experiencing overcrowding as a result of the influx of animals seized from hoarders. Nonetheless, even a cursory check of the news will reveal hoarding cases in which animals are being relocated to shelters in Western countries [16,17,18,19,20].

Similarly, a quick search on the Internet may reveal news of animals being rescued from hoarders and taken to shelters in Eastern countries [21]. According to a recent report based on digital-media coverage, animal hoarding exists in the Russian Federation [22].

Actually, animal hoarding is widely regarded as a Western phenomenon, most likely associated with “pet culture” and capitalism, both of which foster a strong attachment to animals [23], although there is some evidence in the scientific literature that the phenomenon is more widespread [5,22].

Whatever the cause that pushed them into the state, hoarders end up living in filthy conditions of social isolation and extreme personal neglect, suffering profound discomfort because of their situation of environmental inadequacy. It is well known, in fact, that people suffering from hoarding disorder tend to isolate themselves from the outside world and surround themselves with “things” from which they cannot separate themselves, with a proclivity to accumulate until the levels of clutter and lack of living space are reached, making their own daily lives difficult [24,25]. When the accumulation involves animals and, thus, living, sentient beings, the problem becomes more complex and incisive from a welfare standpoint. The inability to care for a large number of animals, confining them in the house or, in some cases, in its outdoor spaces, not only worsens the hygienic and sanitary condition of the environment but also determines the impairment of the physical and sanitary state of the animals themselves, inadequate nutrition, behavioral consequences, and, in the most severe cases, even the death of the weakest individuals [7]. Patronek refers to this type of dysfunctional human–animal relationship as “the third dimension of animal abuse”, in addition to intentional abuse and neglect, because of its detrimental effects on animals, exposing them to significant physical and psychological suffering [26,27]. Several more studies [28,29,30,31] demonstrate that hoarders may unconsciously harm their animals while having a distorted attachment to them. Animal hoarding has been connected to a combination of strong emotional attachments and ineffective, dysfunctional coping mechanisms [31,32]. Some hoarders claim to have a special ability to understand and interact with animals while exhibiting delusional thinking [31,33]. Other situations are created by the behavior of animal owners or carers, particularly of cats, who “cross the line” into hoarding, due to a change in circumstances. This happens to owners or carers of multiple animals whose capacity to care for them is overwhelmed until they lose it due to a variety of factors ranging from uncontrolled reproduction to the onset of physical, emotional, or financial problems.

Assuming that animal hoarding is a very complex syndrome [26,34,35], it is widely stated in the literature that hoarders usually have no awareness of their problem [7,9,31,36,37], do not recognize the distressing conditions of the animals they own [26], and may have underlying cognitive impairments [6]. As a result, the decision to remove the animals from them is common when their situation is discovered. This type of intervention is usually implemented in the most serious cases, in which the animals’ keeping is deemed incompatible with their nature and well-being, if not mistreatment [38]. In situations in which conditions attributable to a criminal offence are detectable, hoarding is prosecuted under animal cruelty laws that allow for seizure and possible forfeiture [26] (In Italy, articles 727 and 544 ter p.c. consider animals as sensitive beings and protect them, respectively, from detention in conditions incompatible with their nature and producing serious suffering and mistreatment, while acknowledging that they are worthy of good welfare. According to Art. 544 sexies p.c., for offences of animal mistreatment, the forfeiture of the animal is always foreseen, unless the animal belongs to a person who is not involved in the offence. Animal cruelty statutes usually demand general intent, while hoarders commonly do not intend to harm their animals; nevertheless, it can often be demonstrated that they deliberately acquire a growing number of animals despite being unable to provide adequate care (see 9, at 21 Section II.A.2.)) A civil approach may be used when animal hoarders are willing to accept help and intervention and appear to be willing to return to normal behavior (see 9, at 21–22 (explaining that civil forfeiture laws have the potential to expedite the animal rescue process)).

It is, however, necessary to have shelters that can accept and care for these animals, which can be difficult challenges because the animals may not adapt and because shelter facilities are often already full and lack enough space to accommodate the large numbers of animals commonly found in hoarders’ homes [39]. Within this framework, ensuring the welfare of these animals becomes challenging, and the question of the welfare of the people who have (mis)kept them up to that point remains open. They, in turn, must be helped to regain a sense of balance in their lives and a healthy relationship with their companion animals [40].

The One Welfare approach [41], which is increasingly being used in conjunction with One Health to work at the interface of human and animal health and welfare, could be adopted to have a positive impact on animals while also addressing poor human conditions. Because positive interactions between humans and animals are an important aspect of it, this approach is appealing when targeted interventions involving the two parties are required, even if they are destined not to resume the relationship later. Furthermore, it is widely acknowledged that animal hoarding is a complex problem that requires the involvement of multiple agencies, ranging from social services to mental health services, environmental health services, and veterinary services [42]. The purpose of this article is to offer insights into how the concept of One Welfare can be applied to address the problem of animal hoarding, as well as the “welfare interests” of the parties involved.

## 2. The One Welfare Approach

There is growing evidence that the condition of well-being extends beyond physical health to mental health and, more broadly, well-being addressed in a multidimensional manner, which must also be considered. This is true for both humans and nonhuman animals and is well summarized in the concept of “One Welfare”, which recently flanked and supplemented the already well-known One Health principle [43]. Both are supported by the link that is established between the welfare of all living things and the ecosystems in which they exist, and the need for an interdisciplinary approach to the study of this interconnectedness [44] and the management of socio-ecological complexity.

Addressing welfare necessitates confronting important (and sometimes contentious) issues in science, health, productivity, economics, politics, and even ethics [45]. As a result, it is critical to have an approach that does not focus on isolated disciplines but connects them like pieces of a puzzle. This composite picture points back to the need for balancing and promoting various welfare interests, which is becoming increasingly apparent in a global context of interconnected ecosystems and societies [46]. Human welfare is important among the various aspects considered, as are the physical and social environments, in addition to the assorted body of animal welfare issues, precisely because of the interlink that exists between the conditions of all life forms that comprise a community.

The interdependence of human and animal conditions stems from a common evolutionary origin and creates a dynamic complexity that requires more than just the human dimension to be considered when addressing the effects of coexistence.

In terms of health, the unifying concept of One Health has long supported policies and programs aimed at improving the health of people, other animals, and the environment [47]. The existence of a relationship between the various human and nonhuman life forms that populate our planet is thus already recognized in terms of health, but it risks being ignored and undersupported in terms of welfare due to the complexity of the area and the fact that evidence is sometimes still developing when it comes to animal mental states [48].

Yet, it has already been established that “animal well-being” and “human well-being” both refer to a state in which “individuals have the psychological, social, and physical resources they require to meet a specific psychological, social, and/or physical challenge” [49]. In fact, in both cases, they evoke a positive mental and emotional state that complements health, allowing one to speak of “quality of life”. The One Welfare concept embraces and draws attention to this connection to break down silos and benefit both humans, animals, and the planet. The One Welfare framework is divided into five sections, which are listed in no particular order of priority in Table 1, according to Garcia Pinillos, 2018 [41]. Section 1 of the One Welfare framework will be considered for the purposes of this article.

## 3. Relationships between Animal Abuse and Human Neglect

Section 1 of the One Welfare framework addresses human–animal interactions that can result in abuse, neglect, and suffering. Building on the research that has already confirmed the link between animal abuse and human abuse, it aims to better understand this connection and highlight its complexity in order to raise awareness of it.

The abuse of vulnerable beings, whether human or animal, implies intentional physical or psychological violence, sometimes with the goal of control or coercion.

Neglect, in contrast, is typically the result of carelessness, indifference, or ignorance; it can also be the result of neglectful behavior that personally affects the perpetrators, who are also careless towards themselves [50]. It implies a failure to provide supervision, basic-needs fulfilment, medical care, and even necessities that the victims cannot provide for themselves [51].

Understanding the link between animal abuse, human violence, and neglect is proposed as a means to identify and potentially prevent incidents of intentional mistreatment directed at humans and society shortly after those directed at animals [52].

As previously stated, the hoarder’s behavior may have illegal traits and, although not necessarily involving malicious intent, may be considered a crime against animals. Indeed, animal neglect results in sacrificing their welfare to the point of causing them severe suffering [41] (see Section 1 of the One Welfare framework). The perception of the welfare of their animals in the minds of the animal hoarders gradually deteriorates, to the point of not realizing the decreasing quality of their condition and convincing themselves that they are well cared for [41] (see Section 1 of the One Welfare framework). However, this does not change the fact that they subject animals to living conditions that are contrary to their nature and, in many cases, intolerable to them.

The environment in which animals are forced to live has a significant impact on their lives and well-being [53]. Therefore, it is critical that animal welfare be included among the non-marginal aspects of social and environmental relevance in the One Welfare approach.

## 4. The Life of an Animal Hoarder

An animal hoarder is generally described in the literature as a person who owns many animals and lives with them in unsanitary conditions [7,26]. Hoarder behavior is defined as following a degenerative course [4], hiding behind a mendacious attitude of “love for animals”, selfish self-servingness [54], a lack of empathy [55], and even elements of criminal relevance, which he or she fails to recognize. The Hoarding of Animals Research Consortium (HARC) has laid out and displayed this characterization in reports professionally documenting the phenomenon of animal hoarding [56].

The following criteria (first proposed by Patronek in 1999, and included in the Fifth Edition of the Diagnostic and Statistical Manual of Mental Disorders) have been identified as being met to frame hoarding behavior, bearing in mind that animal hoarding is characterized as a special manifestation of hoarding disorder [4,12,56]:

- Having a larger than usual number of companion animals;
- The inability to provide minimum acceptable standards of nutrition, sanitation, veterinary care, and hygiene, resulting in illness, injuries (untreated), and even death;
- Denial or minimization of the inability to address the deteriorating condition of the animals (including disease, starvation, or death) and the environment (e.g., severe overcrowding, extremely unsanitary conditions) and avoid the consequences of failure in terms of the human and animal living conditions;
- Persistence in accumulating a collection of animals and/or difficulty in giving up any of them despite progressively deteriorating conditions that are not recognized and the failure to manage them.

Based on their approach, hoarders have been sub-categorized into more specific groups in order to define different forms of hoarding behavior and to help guide intervention strategies, referring to three main categories: overwhelmed caregivers, whose problems in caring for their animals are triggered by a change in circumstances; rescuer hoarders, who tend to actively acquire animals, motivated by a strong sense of a mission to save them from presumed threats and the belief that only they can properly care for the animals they “rescue”; exploiter hoarders, who acquire animals to satisfy personal needs without any real attachment to them, showing indifference to their suffering and denial of any problems [26].

The literature [26] also reports the figures of the “incipient hoarder” and “breeder-hoarder”, whose stories begin with a modest number of animals and then evolve over time until they lose control to the point that they do not know the exact numbers of their animals.

Regarding the category of overwhelmed caregivers, it is worth noting that they do not always fully fit the scientific definition of hoarders, as they may “simply” be owners or carers whose ability to take care of their animals becomes unmanageable for reasons related to a lack of control over their reproduction or to “passive adoptions” (e.g., dogs or cats that they feel have been abandoned near their homes, or stray animals they feel are in need or that other people give to them because they are aware of their empathy for animals). This group may have mental health problems, such as depression, although they usually maintain a degree of awareness of their problems.

Dogs and cats are the most commonly accumulated animals, with an average of 39 animals, but this can reach 100 or more in many cases [57]. As a result, living conditions for both the animals and the humans who share the dwelling become routinely untenable. Inadequate cleaning results in the accumulation of dirt and even animal droppings; the unhealthy environment quickly becomes colonized by parasites, bacteria, mold, and pests, and, in some cases, it is plagued by the presence of dead and unremoved animal carcasses. Toilet facilities are frequently inoperable [57,58]. In the context of a broad medical definition, hoarders are framed under the umbrella of neuroses and personality disorders [59,60,61]. Along with these pathological states, senile diseases such as dementia or Alzheimer’s disease can be included, and memory and attention problems have been documented in hoarders [62]. Hoarders’ behavior can be compared to addictions in which impulse control is impaired [56,59], while self-abandonment is very common, especially in the elderly, who are at risk of malnutrition, poor treatment management, and eviction from the home [4,56].

These deteriorated living conditions do not always affect exploiter hoarders, breeder-hoarders, or overwhelmed owners or carers, as they may keep their animals outside their own homes and demonstrate a moderate ability to care for them.

The problems and inconveniences that threaten the health and safety of hoarders and impair their daily lives also have an impact on those who live with them, members of the surrounding communities, and, more broadly, society as a whole, with reference to the expenses that become necessary for cleaning and pest control or relocation to new housing, which are generally borne by public authorities [7,9,26]. This is true despite the fact that the costs of animal accumulation are frequently overlooked and underestimated, but the few studies that have estimated them report the high levels of the costs involved and generated for governmental and non-governmental institutions, especially when the rescue efforts are in regard to large-scale animal-hoarding cases [11,40,63,64].

The degree to which hoarders perceive and understand their own level of social symptoms and needs varies but tends to be very poor, as confirmed by their general reluctance to cooperate and resistance to change [7,26,40,54,65], which is most likely why the recidivism rate tends to be high, implying that commonly used intervention strategies are significantly ineffective [26,40].

The obsessive drive to hoard animals is usually linked to the need for control over a possession and the desire to maintain it; thus, when others, whether another person or agency, claim to “touch” animals in order to care for them, it commonly becomes highly distressing for the hoarder [66]. It is particularly difficult for the authorities to take control of hoarded animals because people suffering from animal-hoarding syndrome often avoid them, refuse to cooperate, and are very resistant to external interventions, asserting their unique ability to care for their animals properly, despite all evidence to the contrary [4,33]. This behavior is frequently motivated by the distorted sense of responsibility that hoarders feel, which is exacerbated by the form of overattachment that characterizes their dysfunctional relationship with animals [10,31,56].

## 5. The Lives of Hoarded Animals

Animals who are hoarded always have welfare issues. They are, in fact, victims of the hoarder’s (often unconscious) need to support his or her own emotional needs, his or her significant lack of empathy, and his or her misguided sense of treating them well. As a result, their true needs go unmet [26].

Hoarded animals are typically kept in deplorable conditions, such as filth, neglect, malnutrition, parasitism, infectious diseases, or other untreated chronic conditions. Studies examining medical conditions in hoarded dogs and cats have found a variety of infections and diseases, affecting the respiratory and/or gastrointestinal system, skin, ears, and mouth. Many animals are injured and, in a significant number, they developed chronic diseases, such as upper respiratory infections (URIs), the insurgences of which are linked to the lack of treatment of an initial disease, long-lasting stress, and prolonged exposure to an unhealthy, overcrowded environment. Compared to non-hoarded animals, hoarded animals show the effects of a continuum of incidences of unfavorable environments in which they live that exacerbate their diseases. The percentage of animals found dead or in such poor condition that euthanasia is required ranges from 25% to 53% of the total number of animals found [9,26,35,36,67,68]. Furthermore, these animals are deprived of an environment appropriate to their ethology and forced to live in conditions contrary to their nature, resulting in a life of deprivation, pathological states, pain, and suffering. They frequently develop abnormal behaviors as a result of poor socialization, such as fear, sensitivity to touch, separation anxiety, stereotypies, and chronic stress [69]. They almost never receive spay/neuter assistance or veterinary care, and another concern is that the animals’ suffering is prolonged in hoarding conditions. The deficiencies to which they are subjected, as well as a lack of veterinary care and proper social interaction, characterize their entire existence around the hoarder and can lead to slow agony [35]. Furthermore, it has been documented that hoarding can have long-term effects on animals, even after they are removed from the hoarder and placed for adoption with “normal” families [70]. Dogs coming from poor conditions in their early life experiences with humans show socialization problems, exhibiting altered stress responses and behavior abnormalities, such as fear, timidity, and separation-related problems, and they develop attachment bonds with humans, but in altered forms (e.g., with increased attention seeking or “hyperattachment”) [71]. The problem of animal hoarding is so complex that addressing it requires the collaboration of many disciplines and professional figures, including psychologists and social workers [72], sanitation workers [56], veterinarians [55], lawyers [73], and others. This diverse input of expertise and interventions is beneficial not only in addressing all aspects involved but also in preventing recidivism. A holistic approach is thus preferable, and the One Welfare principle can assist in achieving an efficient solution for all stakeholders.

## 6. The Lives of Animals in a Shelter

Animal shelters are designed to accept and protect animals who do not have families to care for them or who have been abandoned for a variety of reasons. They have evolved over time, but the kind and quality of welfare provided to animals is not yet uniform due to the diverse resource availability and, particularly, different statutes and shelter laws that establish standards with differing degrees of detail. Unlike in the past, many modern shelters adopt a no-kill policy and do not euthanize animals as soon as they enter or after a few days if they are not claimed by an owner. Several countries, including Italy, Austria, Germany, the Czech Republic, India, Taiwan, and Costa Rica, have implemented laws that prohibit the euthanasia of healthy or treatable animals in shelters [74].

This type of restriction, however, is not standardized, and in some countries or jurisdictions, healthy and treatable shelter animals are still euthanized if not adopted or reclaimed. In the United States of America, for example, despite the fact that some states (such as California) have adopted the no-kill concept, there is no federal law governing shelter facilities and their procedures; this has resulted in several approaches. Over thirty states have “holding period laws” that determine the minimum period that impounded animals must be kept at a shelter before they can be released or euthanized. Following this period (typically three or five days), the decision on the sort of the animals is usually up to the animal shelter [75].

In addition, there are a number of challenges that affect the lives of animals in shelters, beginning with the fact that the shelter system struggles to provide an adequate standard of animal welfare, which requires many resources, both financially and in terms of shelter staff. Above all, the availability of space to house them, taking into account their individual characteristics as well as the possibilities (or impossibilities) of socialization, is critical [76,77].

The picture of existing shelters is not uniform because the care, management, and regulation differ between facilities. Furthermore, it should be noted that the requirement to keep animals in shelters until they are returned to their original owners, adopted by a new family, die of natural causes, or are euthanized for serious health or behavioral reasons can lead to overcrowding and, as a result, worsening welfare conditions. Shelters, while having the common goal of providing adequate space and care for the accommodation requirements of the animals they house, as well as their nutritional and health needs, are generally designed to house animals temporarily (though some often stay for long periods) and do not have the characteristics of a real home [78,79]. Animals are often confined to a small space and often share it with other individuals, as well as access to food and care resources. The care for each animal is reduced when the facility is understaffed [80].

The quality of life and care in the shelter has an impact on the animals, and for some, it can be especially problematic. This is particularly true for cats, which are greatly affected by stress from various factors in their shelter environment, where they can exhibit signs of fear and antagonistic behaviors [67,81,82,83,84,85,86], and when animals are forced into long-term confinement that reduces animal welfare, inducing a decrease in activity, alterations in exploratory and locomotor behavior and sleep patterns, excessive autogrooming and vocalizations, and anxiety [87]. Furthermore, individual differences in reactions to environmental stress have been observed in some subjects in terms of the time spent in a standing posture vs. lying position, the degree of activity, sleep patterns, attention seeking, the resting time, and social behavior [87,88,89].

In any case, arrival in a new context, placement in inadequate space and often in poor environments, the loss of affective bonds or otherwise habitual relationships with the person or people they know, and the presence of other unfamiliar animals are critical aspects for all animals, in addition to the fact that, in the shelter environment, they are over-stimulated in the auditory, olfactory, and visual senses, activating the stress response [78,90,91,92,93,94,95,96]. This is exacerbated if the facility is not functional, if supervision is inadequate, and if sufficient funding is not available [97]. Even in well-managed refuges, the presence of transient, displaced, and mixed animal populations promotes biological instability, which increases the risk of pathogen exposure [98]. In addition, the effects of sheltering on animal behavior have been documented in the literature, with animals unable to cope successfully with the new environment and often developing reduced behavioral variability, abnormal or stereotyped behaviors, as well as stress-related attitudes, such as lack of responsiveness, altered activity levels, or other specific signs, such as vocalizing, self-grooming, and coprophagy [53,99].

Higher rates of fear toward unfamiliar people, other animals, strange objects, and loud noises are commonly reported in seized hoarded animals. Individual differences range from the refusal to be handled or picked up to hyperattachment and attention-seeking behavior, and from aggression toward strangers and excitability to a tendency to hide and increased fears. In several cases, there is an increase in compulsive and repetitive behaviors, including excessive licking [100].

## 7. The Challenges

The One Welfare approach has not yet been extensively researched in terms of practical applications and spin-offs, and it is interesting to point out what challenges are most likely to be faced by those who carry out their animal welfare work in the field for the purposes of future research. Situations involving vulnerable people who own animals necessitate special measures, such as considering not bringing all their animals to shelters. When animal removal is the only option and the shelter doors are opened to them, challenges arise for both the animals, as to their adaptability, and the shelter staff, who must receive and settle them while ensuring their well-being. The work will be more or less complicated and challenging depending on the number of animals and their relative psycho-physical condition, and because the recovery of their welfare and the resources that must be deployed are dependent on them. A critical point arises when considering addressing the condition of their owners in order to determine whether the situation can be resolved and whether the animals can be provided with new welfare. This point fully reflects the mandate of the One Welfare approach. Finding strategies and making decisions that protect both people and animals is a difficult challenge, especially when the goal is to avoid separation. When the decision to remove the animals is not supported by law because, for example, the situation is not severe enough to warrant actual mistreatment or the people holding the animals have mental or cognitive issues, the ethical question of whether it is acceptable to separate them from their animals or whether alternative solutions should be explored arises.

Alternative solutions are consistent with the concept of One Welfare because any useful solution to avoid separating animals from their owners is preferable when welfare issues are resolvable, as this minimizes stress for both parties and improves their living conditions. It also relieves strain on shelters and avoids the expenditure of the economic and professional resources required to support all the steps involved in removing animals and placing them in a shelter, as well as veterinary care (which may include sterilization costs).

Keeping the One Welfare principle in mind in human–animal interactions would imply considering the welfare of the individual animals directly involved as well as the welfare of the humans involved, which is a significant challenge in the case of animal hoarders, who have large numbers of animals. Moreover, from the One Welfare perspective, the goal of ensuring the welfare of all stakeholders extends beyond those directly concerned, taking into account indirect impacts, such as those on society and the environment. This allows the shelter to fit well into the One Welfare framework because it means that the facility should provide welfare opportunities not only for the animals housed there and the staff members and volunteers who care for them but also for the outside community and the environment surrounding it.

When animals from hoarding environments are to be housed, the criterion of considering their needs creates a challenge because their health and behavior are almost always compromised and require a concentration of effort and attention that may not be reconcilable with routine staff activities (that cannot be neglected for the benefit of other housed animals). Furthermore, animals (usually in large numbers) from hoarders arrive at shelters from cruelty cases of hoarding and, after being seized, are legally framed as “seized” but not yet forfeited. Thus, they are taken away from the owner, who, however, does not lose ownership rights until convicted because animals are classified as property by the legal system. Moreover, seized animals sometimes must be retained as evidence in the prosecution of criminal cases [101]. In all of these circumstances, and especially when a judgment on animal ownership is pending, animals are required to be held at the shelter and cannot be adopted by a new family until the court case is finalized, which could take months or years.

These legal circumstances impose restrictions on shelter workers, who must obtain permission for interventions that are limited by “property rights”, such as spaying/neutering. These animals are not available for adoption as long as the hoarder owns them. This lengthens their stay in shelters, even if they have no specific problems to recover from. Shelter staff is required to be ready to make decisions in these cases, either by activating the authorities to obtain the necessary permits, for example, or by employing a strategy of alternative measures, which can “circumvent” the legal constraints (For example, establishing temporary fostering for families who are aware of the animals’ legal status and are willing to possibly return them if the seizure is not confirmed but who can offer them a better condition (and welfare) than in the shelter in the meantime) and also relieve the number of animals in the facility.

In fact, managing large numbers of animals, many of which are in poor condition and whose mental and physical health must be assessed, is a significant challenge in and of itself.

Examples of how a One Welfare approach might work in animal sheltering, particularly in managing the influx of animals moved from hoarding environments, are still limited in the scientific literature, and do not specifically address the problem of animal hoarding [102,103]. Furthermore, studies that assess the workload that would be created for animal welfare officers and other personnel involved in the practical functions, as well as that describe the impacts of real inter-agency coordination, are still lacking [104]. A proposal could be structured around the following initiatives, involving all stakeholders, both human and non-human.

Integrating this concept into animal welfare programs and policies should be one of the first steps. Animal welfare organizations may be the ones to change their activities while waiting for this to happen at the regulatory level. Programs that assist individuals in the local community in reducing distress and encouraging the prevention of behavioral drift may be explored in the context of combining animal welfare with human well-being. For example, readily usable information on good management practices for dogs or cats could be provided (e.g., through webpages, local newspapers, information days, and visits by trained volunteers to families who have adopted animals), as well as free or low-cost options for spay/neuter and veterinary care, behavioral rehabilitation, and basic supplies, such as leashes, kennels, carriers, and food. When necessary, transportation to veterinary facilities might be arranged, and the compassionate or emergency boarding of animals could be provided when owners fall ill or encounter other challenges. All of this would assist even less affluent and vulnerable people in caring for the welfare of their animals and reducing the emergence of conditions that require their removal.

It is also remarkable that helping animal hoarders also goes in the direction of reducing harm to the community (of neighbors) associated with environmental issues derived from the hoarders’ behavior.

When animals are brought to shelters, staff must be prepared to manage their needs with extreme caution, considering potential health and behavioral issues. A key concern in this regard is the availability of space to properly assign the animals, considering the health isolation demands and socializing issues with the other dogs that already there. One form of the One Welfare approach might recommend, for example, trying to find out whether the hoarder has a referring veterinarian (they often do, even if they do not regularly take their animals to such a professional and try to hide the fact that they have a large number of animals) [4] and liaising with the veterinarian about the health status of at least the animals that have been brought to visit, possibly gaining other useful information from this form of collaboration. In terms of space, networks could be formed with other shelters to share the effort of accommodating animals when they arrive in large numbers, reducing the difficulties of overcrowding for both animals and shelter staff.

From a “human” standpoint, one should also attempt to anticipate needs and propose supporting solutions before crisis situations occur. Case identification and direct assistance can be promoted to this goal as appropriate, from improving access to social and health services to avoiding discriminatory attitudes. Assessing the status of individual animal owners is undoubtedly complex, but it is vital to the protection of individuals who are living in difficult situations and their animals.

One shortcoming in this structure is the lack of resources, which would allow humans and animals to receive the required assistance. This issue could be mitigated in part by the cooperation across agencies and institutions proposed by the One Welfare concept, which is based on the principle of interdisciplinary collaboration [105]. One solution to foster it could be the creation of an authority in charge, which would manage interactions between institutions responsible for public health and animal welfare issues and also serve as a reference for case reporting, accessible not just to agencies but also to the community. Such expertise could also be beneficial in raising awareness among specific agencies in order to establish forms of practical intervention and assistance aimed at individual situations, including, hopefully, in a preventive form, when reports are made, or in finding solutions to relapsing cases. In terms of prevention, veterinarians could play a key role if they are prepared to recognize “problem” owners, identifying the individual’s conduct as well as the condition of the animals brought in to visit (e.g., if indicative of confinement in filthy conditions). They could provide valuable referrals to social service or public health authorities to encourage checks on the person and the state of his or her living environment. The intervention of such agencies may also play a role in the prevention of recidivism related to the hoarder’s psycho-social condition (it should be noted that increased research on the psychological and psychiatric aspects of the problem is required for this purpose, and it is to be expected that the inclusion of the hoarding disorder in the DSM-5 as a distinct disorder will encourage further studies on the specific form of animal hoarding as well). It could also coordinate institutional networking and the sharing of not only information but also best practices among officers.

The method of institutional ethnography proposed by Koralesky et al. [104] may be useful to produce this service. It may provide an overview of present methods and standards, monitor One Welfare activities, and offer insights into new ways of responding to the needs of animals and people, particularly in stressful situations.

One challenging aspect to anticipate is the likelihood of avoiding criminal situations. In fact, the One Welfare approach seeks to protect both animal and human welfare, and in the case of the latter, an individual’s probable psychiatric condition is relevant. A systematic connection between animal welfare agencies and mental health services would facilitate the identification of intermediate solutions between conviction for ill treatment and acknowledgement of the absence of responsibility for conditions including mental impairment.

The possibility of treating hoarders, including counseling interventions and monitoring with the assistance of animal agencies, could create the conditions for imposing strict limits but not prohibitions on animal keeping, favoring conditions and behaviors of these individuals that are not harmful to themselves or their animals [26,40]. The availability of psychotherapeutic interventions, including cognitive–behavioral-oriented measures, may make it possible to deal with the tendency of people with hoarding disorder to neither seek nor accept external intervention in their lives with a chance of success [40], while also overcoming the limitation of veterinary authorities’ involvement, which can only address the animal welfare aspect of the problem. An intensive intervention strategy could incorporate more specific parts of the intervention in the problem at hand, such as practical skills training and educating hoarders in their environments. When it comes to education, the opportunity to educate communities on the phenomenon of animal hoarding and its distinguishing indications should not be overlooked in order to encourage reporting or other types of collaboration.

A not insignificant aspect of the development of this training activity is precisely the involvement of the community, which would be guided to a greater understanding of this behavior and the overcoming of the taboo and stigma towards animal hoarders, and would help to dissolve the stereotype that, for a long time, has prevented it from understanding the cause of the disorder and its diagnosis, ridiculing the condition and also creating a gender bias along with the character of “crazy cat ladies” [24].

## 8. Discussion

Animal hoarding is still under-reported and underestimated because only the most serious, large-scale cases or those resulting in prosecutions are likely to be discovered [9,106,107], and also because of the tendency of those involved to isolate and hide [40,57,64]. When an animal-hoarding situation is discovered, it is critical to “rescue” both the people and the animals involved by providing the necessary counselling or treatment [101,108]. To this end, collaboration among various agencies is beneficial, ranging from social and health services to veterinary services, as well as emergency services, law enforcement, and animal welfare associations. Early and integrated intervention with diverse expertise allows all the humans and animals involved to be helped more effectively and prevents the situation from deteriorating [38,55].

This benefits overall well-being and increases the likelihood of successful rescue intervention. In comparison to the study of object-hoarding disorder [25], there is little information on the effectiveness of strategies used to address animal hoarding [40,109]. Yet, it is a dysfunction that has devastating consequences for all those involved, causing social problems as well as animal welfare issues. A primary goal of municipal public administrations, as well as social and veterinary services, should be to identify a scientific and methodical approach to studying these cases and developing intervention procedures with a focus on prevention.

Prevention should be addressed broadly as avoiding the establishment and consolidation of hoarding mechanisms and their effects and minimizing recurrences in the cases identified and treated. Many human and animal lives could be saved in this manner. It would imply protecting their well-being and avoiding emotional upheaval. Proper care can significantly improve the physical and behavioral conditions of the people and animals involved. Furthermore, the living environment can be kept in a healthy and comfortable condition.

Preventing large numbers of animals from being seized and taken to shelters, where they will struggle to adapt and which may be unsuitable for their delicate mental and physical recovery needs, is especially important after they have suffered for a long time from the deprivation and discomfort of the hoarding environment. Furthermore, veterinary care is not always guaranteed in shelters, and much of animal welfare is dependent on the available economic and professional resources.

In addition to these general considerations, one must consider the wide range of hoarding situations into which these animals are forced, as well as the length of time the conditions of distress have persisted and the level of mental and physical impairment they have reached. The latter varies on an individual basis and, ideally, should be considered when making the best decisions for each animal, taking into account the prediction of how they would fare in the shelter and whether there are any viable alternatives or forms of support to improve their health and well-being.

In addition, the hoarder’s position must be evaluated in terms of legal responsibilities, as the possibility of seizing his or her animals and transporting them to a shelter is dependent on this. Because animals are not inanimate objects, criminal prosecution for abuse and neglect can occur regardless of the individual’s medical state. In this regard, developing synergies among agencies dealing with the assessment of the hoarder’s state in order to evaluate even intermediate or alternative measures, as prosecution may not be the best answer to a mental health issue, may assist the legal resolution of these situations.

The One Welfare approach, as a complement to the One Health approach, may be appropriate as a foundation for addressing the challenges posed by animal hoarding and moving animals to shelters. Although it must be acknowledged that, in many cases, this risky behavior cannot be stopped, integrating existing strategies with the interdisciplinary collaboration fostered by this concept could improve the resolution of hoarding cases.

This method has already been tested in several communities in the United States, Canada, Australia, and Europe [55,109], and it has proven to be an effective tool for approaching hoarders in a way that addresses the various multidimensional aspects of their condition while also earning their trust. Among the practical implications, the prospects of providing social assistance to vulnerable persons and structuring them in a multidisciplinary and even extended form to help stabilize circumstances are highlighted.

Focusing on the plight of hoarders as well as that of the animals while involving the capacities of social services and “animal services”, such as veterinary professionals and animal behavior experts, recognizes that both human and animal welfare are at stake and important and that the well-being of both these categories should be improved. This makes it advisable to consider alternative measures before seizing animals and moving them to shelters.

From this perspective, the ethical value of the One Welfare approach emerges, which seeks to balance all interests in the best way possible. Taking a proactive approach (as One Welfare suggests), recognizing and involving human and animal services, offers the best prospect of viewing hoarded animals not only as harmed animals but also as a symptom of a problem that requires interdisciplinary solutions [109]. Recognizing that a complex response system is needed allows for the development of an organizational culture that would be beneficial to professional involvement as well as to the community. In the case of animal hoarding, intangible factors, such as ethical and cultural factors, as well as pragmatic aspects, such as health and economic impacts, are relevant. There is no doubt that ensuring the welfare of humans and animals, both of which are living and sentient beings with their own interests, the most important of which is not to suffer, is an ethical goal and moral responsibility of a social and civil community.

The attention paid to taking initiatives aimed not only at humans but also at animals, while taking into account the vulnerability of both categories, not only gives the One Welfare approach an ethical value, but it also brings it in line with the current culture, given the role that animals play in modern societies, including donating unconditional and selfless support [110,111].

## 9. Conclusions

The discovery of animal-hoarding situations highlights the need to care for both the vulnerable people at the center of them and the animals who are equally vulnerable. The One Welfare approach, which complements the One Health approach, can be very helpful in determining the best strategy in each of these situations, considering all the interests involved. To date, research has revealed that the condition of animals found in precarious housing environments is not uniform. However, animals are frequently removed and taken to shelters. This action allows them to be immediately removed from an unhealthy environment. Nevertheless, transfer to a shelter has consequences for the animals’ health and welfare, depending on both their adaptability and the functionality of the facility that will house them.

A One Welfare strategy suggests taking advantage of the interdisciplinary collaboration of different agencies and professionals, but most importantly, it emphasizes the opportunity to explore alternatives to the standard solutions wherever possible. These latter must be improved further and enhanced. More research on this topic is recommended, including evaluations of the outcomes of the various forms of interventions investigated and, possibly, taking into account the unique needs encountered in each case. When hoarders’ animals are taken to shelters, it is critical to compare the work processes implemented and their outcomes and then evaluate them from the One Welfare perspectives. This will make it possible to determine whether the challenges posed by the need to manage these situations are on the way to being met positively.

In this regard, collaboration between researchers and professionals dealing jointly with the problem may play a crucial role in collecting information and developing a practice. Future studies could start from collecting the viewpoints of human and social services staff and examining their work when they have to face situations involving people and their animals. The way animal protection officers must operate with human social services could be investigated as well. In both cases, challenges and the need for resources could be identified, and if the One Welfare initiatives are implemented, empirical observations on the effects could be reported. In a successive phase, participatory research might also be explored, following the opportune ethical guidelines, in order to collect information directly from the stakeholders on their compliance with the offered support or on the effects of the alternative measures adopted towards their condition, following the One Welfare approach.

## Figures and Tables

**Table 1:** The One Welfare framework. From R. Garcia Pinillos’s One Welfare: A Framework to Improve Animal Welfare and Human Well-Being. CAB International, 2018 [41].

The One Welfare Framework
Section 1: The connections between animal and human abuse and neglect.
Section 2: The Social Implications of Improved Animal Welfare.
Section 3: Animal Health and Welfare, Human Well-being, Food Security and Sustainability.
Section 4: Assisted Interventions Involving Animals, Humans, and the Environment.
Section 5: Sustainability: Connections Between Biodiversity, the Environment, Animal Welfare, and Human Well-being.
